# Modeling the Spread of Tuberculosis in Semiclosed Communities

**DOI:** 10.1155/2013/648291

**Published:** 2013-05-09

**Authors:** Mauricio Herrera, Paul Bosch, Manuel Nájera, Ximena Aguilera

**Affiliations:** ^1^Facultad de Ingeniería, Universidad del Desarrollo, Santiago 7620001, Chile; ^2^Facultad de Ingeniería, Universidad Diego Portales, Ejército 441, Santiago 8370179, Chile; ^3^CEPS, Facultad de Medicina, Clínica Alemana, Universidad del Desarrollo, Santiago 7710162, Chile

## Abstract

We address the problem of long-term dynamics of tuberculosis (TB) and latent tuberculosis (LTB) in semiclosed communities. These communities are congregate settings with the potential for sustained daily contact for weeks, months, and even years between their members. Basic examples of these communities are prisons, but certain urban/rural communities, some schools, among others could possibly fit well into this definition. These communities present a sort of ideal conditions for TB spread. In order to describe key relevant dynamics of the disease in these communities, we consider a five compartments SEIR model with five possible routes toward TB infection: primary infection after a contact with infected and infectious individuals (fast TB), endogenous reactivation after a period of latency (slow TB), relapse by natural causes after a cure, exogenous reinfection of latently infected, and exogenous reinfection of recovered individuals. We discuss the possible existence of multiple endemic equilibrium states and the role that the two types of exogenous reinfections in the long-term dynamics of the disease could play.

## 1. Introduction

Dynamics of tuberculosis (TB) spread has been the subject of a considerable body of theoretical and mathematical work. For review, see, for example, [1, 2] and references therein. The choice of a particular model is strongly connected to the questions we want to answer, and in the present work we will address the problem of long-term dynamics of tuberculosis and latent tuberculosis (LTB) in semiclosed communities.

For semiclosed communities we mean not strictly closed communities with certain mobility of their members out or into the community, with a recruitment of new members and departure of others. But, essentially these communities are congregate settings with the potential for sustained daily contact for weeks, months, and even years between community members. Basic examples of these communities are prisons, but certain urban/rural communities, schools, among others could possibly fit well into this general definition. These communities present a sort of ideal conditions for frequent TB outbreaks, enhanced TB transmission, and accelerated spread of the disease.

The basic characteristics of such settings including the possibility of high concentrations of infectious individuals and immunodeficient hosts, improper precautions taken for protection, delay in diagnosis, sustained contact with the index case, and inadequate ventilation and/or overcrowding make them well suited for TB transmission, creating this way genuine high transmission pockets of TB inserted in the general population [3, 4].

In fact, prisons are especially high burden communities, in which incidence and prevalence of TB are very high, and consequently the frequency of infections and reinfections considerably increases in comparison with population at large; see the works by Chiang and Riley [5] and by Baussano et al. [6].

Studying the dynamics of the TB spread in semiclosed communities is an interesting and significant topic by itself; however, there is an important phenomenon due to which the study of these types of communities is essential in the context of TB spread. This phenomenon has been called the *Reservoir Effect* [6, 7]. Indeed, semiclosed communities such as prisons represent a reservoir for disease transmission to the population at large and should be a source of public concern. For example, TB infection may spread into the general population through prison staff, visitors, and close contacts of released prisoners. The transmission dynamics between prisoners and the general population [6], together with immigration from developing countries with high prevalence of TB [8, 9], has been hypothesized to play a key role in driving overall population-level TB incidence, prevalence, and mortality rates.

In a recent work [4] the authors have even gone further in relation to this effect and have named these communities *Institutional Amplifiers of TB Propagation*. Some examples of communities given by these authors are poor hospitals in which dozens of patients share poorly ventilated communal rooms, crowded prison cell blocks, and mining barracks among others.

The transmission and progression of TB infection has been relatively well understood on a population scale. Generally, it is assumed that once an individual is infected with TB, he or she is immune from further infection events. Moreover, it was proposed what came to be known as the *unitary concept of pathogenesis* [10], which states that TB always begins with primary infection, and subsequent episodes of active TB are due to reactivation of dormant bacilli from this primary infection. However, a persistent evidence has recently been shown (see [5] for a review) that the paths to TB infection are not as linear as was suggested by the unitary concept of pathogenesis. The availability of individual, strain-specific infection histories (see, e.g., [11–13]) has made it clear that exogenous reinfection in people with previously documented TB infection does occur. The important question is whether reinfection occurs commonly enough to have an effect on the overall infection dynamics of the population [14].The relative importance of these pathways to the development of active disease has significant implications for treatment and control strategies, most notably in deciding whether latently infected and treated individuals are at risk of reinfection [15].

Several authors [15–20] have declared that exogenous reinfection plays an important role in the disease progression and that the inhalation of tubercle bacilli by persons who have had a primary TB infection previously for more than five years represents an increasing risk to develop active TB soon after reinfection. A study from South Africa [21] has demonstrated that the rate of reinfection by TB after successful treatment could be higher than the rate of new TB infections. In this study the reinfection rate after successful treatment was estimated at 2.2 per 100 person-years, which was approximately seven times the crude incidence rate (313 per 100 000 population per year) and approximately four times the age-adjusted incidence rate of new TB (515 per 100 000 population per year). So, ignoring exogenous reinfection when modeling TB spread in high-incidence and high-prevalence community setting such as semiclosed communities has been seen to be inappropriate. (Henao-Tamayo et al. in [22] recently published a mouse model of TB reinfection that could help to explain immunological aspects of reinfection risk in high-incidence areas.) 

We will use an SEIR standard compartmental model; see for example the works by Blower et al. [23] and more recently by Liao et al. [24] with some modifications explained bellow that turn out to be quite useful in the study of the particularities of TB spread at this type of communities. This model assumes that the population in the community is homogeneous that it does not consider the heterogeneities in the social structure between community members, and it is based on the so-called *mass action* or *fully mixing approximation*. This means that individuals with whom a susceptible individual has contact are chosen at random from the whole community. It is also assumed that all individuals have approximately the same number of contacts in the same time and that all contacts transmit the disease with the same probability.

The model we use in this work takes into account the following relevant facts in the context of semiclosed communities.

1. The overcrowding in the community can increase (compared to what occurs in the population at large) the likelihood of exogenous reinfection due to repeated contacts with active infected individuals. That is, besides primary infection the model considers the possible reinfection of individuals with LTB (individuals who are assumed to be asymptomatic and noninfectious but capable of progressing to active TB) and recovered individuals (individuals who have been treated for TB in the past and been declared cured). If latently infected or recovered individuals remain in the community, they could be infected again.
2. At present, it is not completely clear whether in all cases previous infections with *Mycobacterium TB* with or without subsequent recovery offer some protection that could be translated into a reduced susceptibility to reinfection [5, 21, 22, 25]. So, we will be open at exploring different situations with regard to this fact in the model.
3. Poor nutrition, immunodepression, and other diseases increase the likelihood of accelerated progression to active TB.

We will see that considering exogenous reinfection to describe TB spread produces a richer and more complex dynamics than the one observed in previous models (see e.g., [23, 25, 26]). In particular, unlike the model published by Feng et al. in [26], which uses a single parameter for exogenous reinfection, our model uses two parameters related to two possible reinfections (reinfection of latently infected and reinfection of recovered individuals).

## 2. Basic Epidemiology of TB Sources and Probability of Infection in Semiclosed Communities

The risk of infection with *Mycobacterium tuberculosis*, the bacterium causing TB, depends mainly on two factors: first, significant exposure to a source of infection and second, the probability of getting infection if there is exposure.

TB is mostly transmitted through the air; tubercle bacilli, that depends on host and agent factors, is distributed in tiny liquid droplets that are produced when someone with clinical or active TB coughs, sneezes, spits, or speaks, allowing infected individual to infect others. In closed places the bacteria are expelled into a finite volume of air unless there is ventilation, see [27]. In these conditions they may remain viable and suspended in the air for a prolonged period of time. But, the number of bacilli excreted by most persons with active pulmonary TB is relatively small [16], so the probability of TB transmission per contact, per unit of time is in general quite low. The risk of infection is very small during a single encounter with an infectious individual [28]. However, the probability of TB transmission can be enhanced by systematic and long exposure of susceptible individuals to particular infectious individuals.

The risk of TB transmission is particularly high in settings with poorly ventilated areas (places with reduced air volume per occupant, with ventilation systems which recirculate the air, or with poorly filtered air exchanges) and/or closed areas in which people are in close and frequent contact. Closed regime prisons are examples of these high-risk areas. In effect, the occurrence of TB in prisons for example is usually reported to be much higher than the average levels reported for the corresponding general population [6].

Although most exposed individuals develop an effective immune response to the initial infection [17], there is another factor that raises the chances of TB contagion, the fact that TB is an opportunistic disease. Indeed, infected individuals with weakened immune systems are at significant risk of developing clinical TB disease (active TB). High TB prevalence is therefore observed in individuals with HIV infection, poor nutritional status, alcoholism, drug abuse, concurrence of other pathology, and psychological stress decrease immune response levels. These conditions occur frequently in imprisoned peoples.

TB is usually described as a slow disease because of its long and variable period of latency and because of its short and relatively narrow infectious period distribution. Long periods of latency (inactive TB or latent TB or LTB) imply that new cases of infection are not clinically noticeable and therefore remain unobserved for a period of time. Immune response of susceptible individuals can restrict proliferation of the bacilli leading to what seems to be long-lasting partial immunity against reinfection or a response capable of stopping the progression from LTB to active TB.

Exposed individuals may remain in the latent stage for long and variable periods of time. In fact, it often happens that the host dies without ever developing active TB. The progression from latent to active TB is uncommon in the population at large. It is estimated that only about 5 to 10 percent of LTB individuals develop clinical or active TB [16], but due to the above described extreme conditions at semiclosed communities such as prisons, persons lived in these communities may be at risk of rapid progression from LTB to active TB following recent infection or reactivation of latent infection, or reinfection, see [6].

Some additional known epidemiological facts to be considered for TB disease are the following.

1. Most of the secondary infections generated by an infected individual do take place within the first months following TB activation [29].
2. In the work by Styblo [16] it was noted that nearly 60 percent of the new cases arose during the first year following infection, while the cumulative number of cases generated over the first five years after infection accounted for nearly 95 percent of the total observed cases. People ill with TB can infect up to 10–15 other people through close contact over the course of a year [30].
3. Case fatality among untreated pulmonary TB cases is around 66.6 percent [30].
4. Recovered individuals, naturally or from treatment, may develop active TB again, a phenomenon known as TB relapse. (Recurrent cases (formerly relapse cases) have been treated for TB in the past and been declared successfully treated (cured/treatment completed) at the end of their treatment regimen. Recurrent cases include relapses due to the same *Mycobacterium tuberculosis* strain as for the previous episode as well as new episodes of TB due to reinfection.)
5. Individuals with LTB may progress to active TB due to reexposure and reinfection. The extent to which latent tuberculosis infection could reduce the risk of progressive disease following reinfection is not known [31].

## 3. A Compartmental Model for the TB Spread

**Figure 1:**
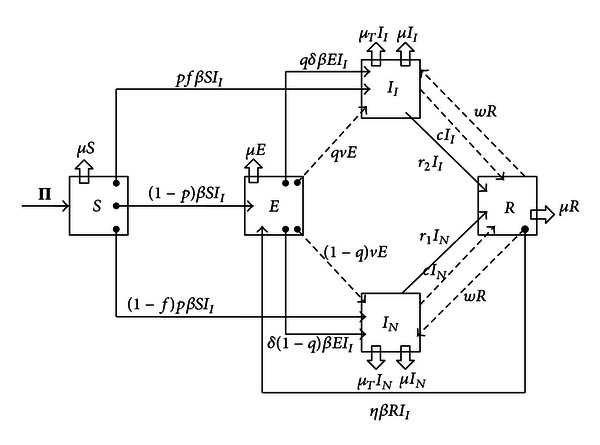
Flow chart of TB compartmental model.

In order to describe key relevant dynamics in the study of the TB spread in semiclosed communities, we consider five compartments SEIR model represented in Figure 1.

The compartments are uninfected individuals (susceptible), the *S* class; the latent class *E*, that is, individuals who are assumed to be asymptomatic and noninfectious but capable of progressing to the clinical disease or active TB; the infectious class *I* is subdivided into two subclasses: (a) infected and infectious individuals *I*_*I*_ and (b) infected and noninfectious individuals *I*_*N*_; and the *R* class of recovered by treatment, self cure, or quarantine.

Every individual in the *E*, *I*_*I*_, and *I*_*N*_ classes is considered infected. There are five possible routes toward TB infection according to this model: primary infection after a contact with infected and infectious individuals (fast TB), endogenous reactivation after a period of latency (slow TB), relapse by natural causes after a cure, exogenous reinfection of latently infected, and exogenous reinfection of recovered individuals.

The *f* and *q* are probability of developing infectious TB if one develops fast and slow TB, respectively, 2*w* is the relapse rate to active TB. Uninfected individuals are recruited at the rate Π, and *μ* is the natural mortality rate. Individuals with TB experience a death rate *μ*_*T*_ due to TB infection.

After infection, a fraction *p* of individuals progresses to disease relatively soon (i.e., within the first two years) after infection; the remaining fraction 1 − *p* of infected individuals become latently infected. Of newly infected individuals who thus progress quickly to disease, *f* represents the fraction that develops infectious disease, and 1 − *f* represents the fraction that develops noninfectious disease.

The *E* class, latently infected individuals, does not shed bacilli and is not infective to others. In some latently infected individuals, the infection remains latent and it may persist for life. But, in a small minority of latently infected individuals, reactivation of the latent infection leads to the disease. The coefficients *r*_1_ and *r*_2_ denote the treatment rates for infected and infectious individuals *I*_*I*_ class and infected and noninfectious individuals *I*_*N*_ class, respectively. The model does not consider unsuccessful treatments.

The parameter *β* is the primary TB transmission rate; this parameter summarizes socioeconomic and environmental factors that affect primary TB transmission. We assume that transmission rates are determined by broad demographic and social contexts, as well as by characteristics of both the transmitter and recipient (i.e., the number, viability, and virulence of the organisms within sputum droplet nuclei, immune status of the recipient, etc.)

TB transmission rate in case of reinfection might be different than the transmission rate of primary infection. The quantities that take into account these differences in case of reinfection of latently infected individuals and reinfection of recovered individuals are given by the dimensionless parameters *δ* and *η*, respectively. The parameter *δ* is the proportion in TB transmission due to exogenous reinfection of latently infected individuals, and *η* is the proportion in TB transmission due to exogenous reinfection of recovered individuals. Thus, *δβ* is the exogenous reinfection rate of latently infected, and *ηβ* is the exogenous reinfection rate of recovered individuals.

A conservative point of view will consider that *biologically plausible* values for the reinfection parameters *δ* and *η* are given within the intervals 0 ≤ *δ* ≤ 1, 0 ≤ *η* ≤ 1. In this case, the parameters *δ* and *η* can be interpreted as factors reducing the risk of reinfection of an individual who has previously been infected and has acquired some degree of protective immunity. However, studies on genetic predisposition [22] or in communities with cases as those reported in [21] have gathered some evidence that in certain situations there may be some increased susceptibility to reinfection. Therefore, we are willing to explore in the next sections other mathematical possibilities where the reinfection parameters can take even less usual values *δ* > 1 and *η* > 1.

However, recurrent TB due to endogenous reactivation (relapse) and exogenous reinfection could be clinically indistinguishable [32]; they are independent events. For this reason, beside primary infection we will include in the model the possibility of endogenous reactivation and exogenous reinfection as different way toward infection. So, we have the following.

1. TB due to the endogenous reactivation of primary infection (exacerbation of an old infection) is considered in the model by the terms *qνE* and (1 − *q*)*νE*.
2. TB due to reactivation of primary infection induced by exogenous reinfection is considered by the terms *δqβEI*_*I*_ and *δ*(1 − *q*)*βEI*_*I*_.
3. Recurrent TB due to exogenous reinfection after a cure or treatment is described by the term *ηβ* *I*_*I*_ *R*.

The parameters of the model, its descriptions, and its units are given in Table 1.

All these considerations give us the following system of equations:

(1) dSdt=Π−βSII−μS,dEdt=(1−p)βSII+ηβRII−(ν+μ)E−δβEII,dIIdt=fpβSII+qνE+wR−(μ+μT+c+r1)II+δqβEII,dINdt=(1−f)pβSII+(1−q)νE+wR−(μ+μT+c+r2)IN+δ(1−q)βIIE,dRdt=c(II+IN)−(2w+μ)R−ηβRII+r1II+r2IN.

Adding all the equations in (1) together, we have

(2) $$\begin{array}{c} \frac{dN}{dt}=-\mu N-\mu_{T}\left(I_{I}+I_{N}\right)+\Pi , \end{array}$$

where *N* = *S* + *E* + *I*_*I*_ + *I*_*N*_ + *R* represents the total number of the population, and the region

(3) $$\begin{array}{c} D=\left\{\left(S,E,I_{I},I_{N},R\right)\in {\mathbb{R}}_{+}^{5}:S+E+I_{I}+I_{N}+R\leq \frac{\Pi }{\mu }\right\} \end{array}$$

is positively invariant of system (1).

It is a common practice in epidemic research to introduce the basic reproduction number *R*_0_, defined as the average number of secondary infections produced by an infected individual in a completely susceptible population, as the measure for the epidemic thresholds, if *R*_0_ > 1 an epidemic will arise.

We have calculated *R*_0_ for this model using the *Next Generation Method* [35] and it is given by

(4) R0=βΠ((hfp+(1−p)νq)(ab−mw)+mw(h(1−f)p+(1−p)ν(1−q)))   ×(μha(ab−gw−mw))−1,

where

(5) $$\begin{array}{c} a=\mu +\mu_{T}+c,\\ b=2w+\mu ,\\ h=\nu +\mu ,\\ g=r_{1}+c,\\ m=r_{2}+c. \end{array}$$

### 3.1. Steady-State Solutions

In order to find steady-state solutions for (1) we have to solve the following system of equations:

(6) 0=Π−βSII−μS,0=(1−p)βSII+ηβRII−(ν+μ)E−δβEII,0=fpβSII+qνE+wR−(μ+μT+c+r1)II+δqβEII,0=(1−f)pβSII+(1−q)νE+wR  −(μ+μT+c+r2)IN+δ(1−q)βIIE,0=c(II+IN)−(2  w+μ)R−ηβRII+r1II+r2IN.

Solving system (6) with respect to *I*_*I*_ we have the following equation:

(7) $$\begin{array}{c} I_{I}\left(AI_{I}^{3}+BI_{I}^{2}+CI_{I}+D\right)=0. \end{array}$$

The coefficients of (7) are all expressed as functions of the parameters listed in Table 1. However, these expressions are too long to be written here. See Appendix A for explicit forms of the coefficients.

#### 3.1.1. Disease-Free Equilibrium

For *I*_*I*_ = 0 we get the disease-free steady-state solution:

(8) $$\begin{array}{c} P_{0}=\left(S_{0},E_{0},I_{I_{0}},I_{N_{0}},R_{0}\right)=\left(\frac{\Pi }{\mu },0,0,0,0\right). \end{array}$$

**Lemma 1**

The disease-free steady-state solution *P*_0_ is locally asymptotic stable for *R*_0_ < 1.

**Proof**

The Jacobian matrix of system (6) evaluated in *P*_0_ = ((Π/*μ*), 0,0, 0,0) is

(9) $$\begin{array}{c} J=\left[\begin{array}{ccccc} -\mu & 0 & -\frac{\beta \;\Pi }{\mu } & 0 & 0\\ 0 & -\nu -\mu & \frac{\left(1-p\right)\beta \Pi }{\mu } & 0 & 0\\ 0 & q\nu & \frac{fp\beta \Pi }{\mu }-\mu -\mu_{T}-c-r_{1} & 0 & w\\ 0 & \left(1-q\right)\nu & \frac{\left(1-f\right)p\beta \Pi }{\mu } & -\mu -\mu_{T}-c-r_{2} & w\\ 0 & 0 & r_{1}+c & r_{2}+c & -2w-\mu \end{array}\right]. \end{array}$$

The characteristic equation for *J* have the form

(10) $$\begin{array}{c} \left(\lambda +\mu \right)\left(\lambda^{4}+a_{3}\lambda^{3}+a_{2}\lambda^{2}+a_{1}\lambda +a_{0}\right)=0. \end{array}$$

Given the polynomial

(11) $$\begin{array}{c} P\left(\lambda \right)=\lambda^{4}+a_{3}\lambda^{3}+a_{2}\lambda^{2}+a_{1}\lambda +a_{0}=0, \end{array}$$

in the special case when *a*_1_, *a*_2_, *a*_3_ > 0, 3 roots of the polynomial *P*(*λ*) have negative real part and if

- *a*_0_ = 0, the 4th root, or largest eigenvalue, is zero,
- *a*_0_ > 0, all eigenvalues are negative,
- *a*_0_ < 0, the largest eigenvalue has positive real part.

Thus, the stability of disease-free steady-state solution is determined solely by the sign of the constant term *a*_0_ of the polynomial *P*(*λ*) [36].

The coefficients *a*_0_, *a*_1_, *a*_2_, *a*_3_ are all decreasing functions of *β* and they are linear functions with respect to the parameter *β*, so they all take the general form *a*_*i*_(*β*) = −*A*_*i*_ *β* + *B*_*i*_ with *i* = 0,1, 2,3 and *A*_*i*_, *B*_*i*_ > 0. We can define *β*_*i*_ = *B*_*i*_/*A*_*i*_. It is easy to see that for *β* < *β*_*i*_ we have *a*_*i*_(*β*) > 0.

For example,

(12) $$\begin{array}{c} a_{3}\left(\beta \right)=-\frac{fp\beta \;\Pi }{\mu }+\nu +3\mu +\mu_{T}+c+r_{2}+2w,\\ \beta_{3}=\frac{\left(\nu +3\mu +\mu_{T}+c+r_{2}+2w\right)\mu }{fp\Pi }. \end{array}$$

By straightforward calculations and reminding that the coefficients *a*_*i*_ are decreasing functions of *β*, we found that

(13) $$\begin{array}{c} a_{2}\left(\beta_{3}\right)<0\Rightarrow \beta_{2}<\beta_{3},\\ a_{1}\left(\beta_{2}\right)<0\Rightarrow \beta_{1}<\beta_{2},\\ a_{0}\left(\beta_{1}\right)<0\Rightarrow \beta_{0}<\beta_{1}. \end{array}$$

This way, *β*_0_ < *β*_1_ < *β*_2_ < *β*_3_ and if we take *β* < *β*_0_, all the coefficients *a*_*i*_ are positive. But, for *R*_0_ < 1, we can see that the condition *β* < *β*_0_ is fulfilled. Indeed, the constant term *a*_0_ of the polynomial *P*(*λ*) can be written as

(14) $$\begin{array}{c} a_{0}\left(\beta \right)=-A_{0}\beta +B_{0}=B_{0}\left(1-\frac{A_{0}\beta }{B_{0}}\right)=B_{0}\left(1-R_{0}\right). \end{array}$$

Using this form for the coefficient *a*_0_ we can see that if *R*_0_ < 1, then *a*_0_(*β*) > 0 so *β* < *β*_0_.

**Remark 2**

For *R*_0_ > 1 we have *a*_0_ < 0, and the disease-free steady-state solution is unstable. Indeed, if *λ*_1_, *λ*_2_, *λ*_3_, and *λ*_4_ are the roots of polynomial *P*(*λ*) = 0, we have *λ*_1_ *λ*_2_ *λ*_3_ *λ*_4_ = *a*_0_ < 0, and it is impossible that the four roots have negative real part.

#### 3.1.2. Endemic Equilibria

From (7) we get that nontrivial solutions are possible if

(15) $$\begin{array}{c} AI_{I}^{3}+BI_{I}^{2}+CI_{I}+D=0. \end{array}$$

The explicit expressions for the coefficients *A* and *D* are

(16) $$\begin{array}{c} A=\beta^{3}\delta \sigma \left(\mu +\mu_{T}\right)\left(\mu +\mu_{T}+c+\left(1-q\right)r_{1}+qr_{2}\right)>0, \end{array}$$

(17) D=h([ab+wr2+r1(w+μ)](μ+μt) +  μm(r1+a))(1−R0),

where parameters *a*, *b*, *h*, and *m* are defined as in (5).

The coefficients *B* and *C* can be written in the following general form:

(18) $$\begin{array}{c} B=\beta^{2}f_{B}\left(\beta \right),\\ C=\beta f_{C}\left(\beta \right), \end{array}$$

where

(19) $$\begin{array}{c} f_{B}\left(\beta \right)=-B_{1}\beta +B_{2},\\ f_{C}\left(\beta \right)=-C_{1}\beta +C_{2}. \end{array}$$

The coefficients {*B*}_*i*=1,2_ and {*C*}_*i*=1,2_ are all positive and depend on the parameters given in Table 1. See Appendix A for the explicit form of these coefficients.

Changes in the signs of the coefficient *B* and *C* as function of transmission rate *β* can be explained using the above defined functions *f*_*B*_(*β*) and *f*_*C*_(*β*), respectively. The functions *f*_*B*_(*β*) and *f*_*C*_(*β*) both are linear and decreasing functions of *β*.

Consider the polynomial function

(20) $$\begin{array}{c} P\left(x\right)=Ax^{3}+Bx^{2}+Cx+D. \end{array}$$

From (17) we can see that for *R*_0_ > 1 the coefficient *D* is negative, so we have *P*(0) = *D* < 0. On the other hand, because the coefficient *A* is always positive, there must be a value x* such that, for *x* > x*, it holds that *P*(*x*) > 0. Since function *P*(*x*) is continuous, this implies the existence of solution for the equation *P*(*x*) = 0.

To determine how many possible endemic states arise, we consider the derivative *P*′(*x*) = 3*Ax* ^2^ + 2*Bx* + *C*, and then we analyse the following cases.

(1) If Δ = *B* ^2^ − 3*AC* ≤ 0, *P*′(*x*) ≥ 0 for all *x*, then *P*(*x*) is monotonically increasing function and we have a unique solution, that is, a unique endemic equilibrium.
(2) If Δ ≥ 0, we have solutions of the equation *P*′(*x*) = 0 given by
  (21) $$\begin{array}{c} x_{2,1}=\frac{-B\pm \sqrt{B^{2}-3AC}}{3A} \end{array}$$

and *P*′(*x*) ≥ 0 for all *x* ≥ *x*_2_ and *x* ≤ *x*_1_. So, we need to consider the positions of the roots *x*_1_ and *x*_2_ in the real line. We have the following possible cases.

- If *C* ≤ 0, then for both cases *B* ≥ 0 and *B* < 0, we have *x*_1_ < 0, *x*_2_ > 0 and *P*′(*x*) > 0 for all *x* ≥ *x*_2_ ≥ 0. Given that *P*(0) = *D* < 0, this implies the existence of a unique endemic equilibrium.
- If *B* ≥ 0 and *C* ≥ 0, then both roots *x*_1_ and *x*_2_ are negative and *P*′(*x*) > 0 for all *x* ≥ 0.
- If *B* < 0 and *C* > 0, then both roots *x*_1_ and *x*_2_ are positive and we have the possibility of multiple endemic equilibria. This is a necessary condition, but not sufficient. It must be fulfilled also that *P*(*x*_1_) ≥ 0.

Let *β*_*B*_ be the value of *β* such that *f*_*B*_(*β*_*B*_) = 0 and *β*_*C*_ the value of *β* such that *f*_*C*_(*β*_*C*_) = 0. Moreover, let *β*_*R*_0__ be the value for which the basic reproduction number *R*_0_ is equal to one (the value of *β* such that coefficient *D* becomes zero).

**Lemma 3**

If the condition *β*_*R*_0__ < *β*_*C*_ < *β*_*B*_ is met, then system (1) has a unique endemic equilibrium for all *β* > *β*_*R*_0__ (Table 3).

**Proof**

Using similar arguments to those used in the proof of Lemma 1,  we have, given the condition *β*_*R*_0__ < *β*_*C*_ < *β*_*B*_, that for all values of *β* such that *β* < *β*_*R*_0__, all polynomial coefficients are positive; therefore, all solutions of the polynomial are negative and there is no endemic equilibrium (positive epidemiologically meaningful solution).

For *β*_*R*_0__ < *β* < *β*_*C*_ the coefficients *B* and *C* are both positive, while the coefficient *D* is negative; therefore, appears only one positive solution of the polynomial (the greatest one), so we have a unique endemic equilibrium.

For *β*_*C*_ < *β* < *β*_*B*_, the coefficient *C* is negative and *B* is positive. According to the cases studied above we have in this situation a unique endemic equilibrium.

Finally, for *β* > *β*_*B*_ the coefficients *B* and *C* are both negative, and according to the study of cases given above we also have a unique positive solution or endemic equilibrium.

Let us first consider biologically plausible values for the reinfection parameters *η* and *δ*, that is, values within the intervals 0 ≤ *δ* ≤ 1, 0 ≤ *η* ≤ 1. This means that the likelihood of both variants of reinfections is no greater than the likelihood of primary TB. So, we are considering here partial immunity after a primary TB infection.

**Lemma 4**

For biologically plausible values (*δ*, *η*)∈[0,1]×[0,1] system (1) fulfils the condition *β*_*R*_0__ < *β*_*C*_ < *β*_*B*_.

**Proof**

Using straightforward but cumbersome calculations (we use a symbolic software for this task), we were able to prove that if we consider all parameters positive (as it is the case) and taking into account biologically plausible values (*δ*, *η*) ∈ [0,1] × [0,1], then *f*_*B*_(*β*_*C*_) > 0 and *D*(*β*_*B*_) > 0 and it is easy to see that these inequalities are equivalent to *β*_*R*_0__ < *β*_*C*_ < *β*_*B*_.

**Figure 2:**
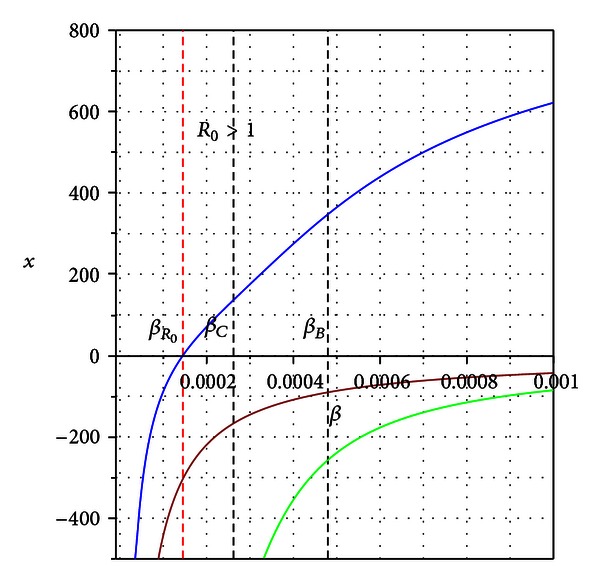
Bifurcation diagram (solution *x* of polynomial (20) versus *β*) for the condition *β*_*R*_0__ < *β*_*C*_ < *β*_*B*_. *β*_*R*_0__ is the bifurcation value. The blue branch in the graph is a stable endemic equilibrium which appears for *R*_0_ > 1.

We have proven that the condition *β*_*R*_0__ < *β*_*C*_ < *β*_*B*_ implies that the system can only realize two epidemiologically meaningful (nonnegative) equilibrium states. Indeed, if we consider the disease transmission rate *β* as a bifurcation parameter for (1), then we can see that the system experiences a *transcritical bifurcation* at *β* = *β*_*R*_0__, that is, when *R*_0_ = 1 (see Figure 2). If the condition *β*_*R*_0__ < *β*_*C*_ < *β*_*B*_ is met, the system has a single steady-state solution, corresponding to zero prevalence and elimination of the TB epidemic for *β* < *β*_*R*_0__, that is, *R*_0_ < 1, and two equilibrium states corresponding to endemic TB and zero prevalence when *β* > *β*_*R*_0__, that is, *R*_0_ > 1. Moreover, according to Lemma 4 this condition is fulfilled in the biologically plausible domain for exogenous reinfection parameters (*δ*, *η*)∈[0,1]×[0,1]. This case is summarized in Table 2.

From Table 2 we can see that although the signs of the polynomial coefficients may change, other new biologically meaningful solutions (nonnegative solutions) do not arise in this case. The system can only display the presence of two equilibrium states: disease-free or a unique endemic equilibrium.

The basic reproduction number *R*_0_ in this case explains well the appearance of the *transcritical bifurcation*, that is, when a unique endemic state arises and the disease-free equilibrium becomes unstable (see blue line in Figure 2).

However, the change in signs of the polynomial coefficients modifies the qualitative type of the equilibria. This fact is shown in Figures 5 and 7 illustrating the existence of *focus* or *node* type steady-sate solutions. These different types of equilibria as we will see in the next section cannot be explained using solely the reproduction number *R*_0_.

In the next section we will explore numerically the parametric space of system (1), looking for different qualitative dynamics of TB epidemics. We will discuss in more detail how dynamics depends on the parameters given in Table 1, especially on the transmission rate *β*, which will be used as bifurcation parameter for the model.

Let us consider here briefly two examples of parametric regimes for the model in order to illustrate the possibility to encounter a more complex dynamics, which cannot be solely explained by changes in the value of the basic reproduction number *R*_0_.

*Example I.* Suppose *β* = *β*_*R*_0__, this implies that *R*_0_ = 1 and *D* = 0; therefore, we have the equation:

(22) P(x)=Ax3+Bx2+Cx=x(Ax2+Bx2+C)=0.

It is easy to see that besides zero solution, if *B* < 0, *C* > 0 and *B* ^2^ − 4*AC* > 0, (22) has two positive solutions *x*_1_ and *x*_2_. So, we have in this case three nonnegative equilibria for the system.

The condition *B* < 0 for *β* = *β*_*R*_0__ means *f*_*B*_(*β*_*R*_0__) < 0, and this in turn implies that *β*_*B*_ < *β*_*R*_0__. On the other hand, the condition *C* > 0 implies *f*_*C*_(*β*_*R*_0__) > 0 and therefore *β*_*R*_0__ < *β*_*C*_.

Gathering both inequalities we can conclude that if *β*_*B*_ < *β*_*R*_0__ < *β*_*C*_, then the system has the possibility of multiple equilibria.

**Figure 3:**
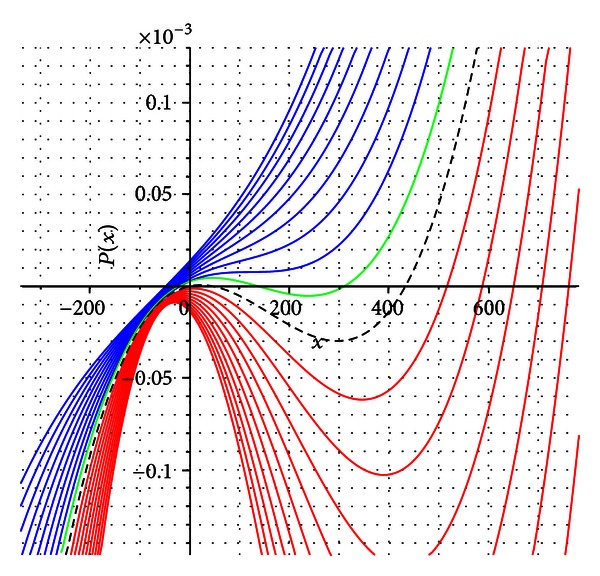
Polynomial *P*(*x*) for different values of *β* with the condition *β*_*B*_ < *β*_*R*_0__ < *β*_*C*_. The graphs were obtained for values of *δ* = 3.0 and *η* = 2.2. The dashed black line indicates the case *β* = *β*_*R*_0__. The figure shows the existence of multiple equilibria.

Since the coefficients *A* and *B* are both continuous functions of *β*, we can always find a *ϵ* neighbourhood of *β*_*R*_0__, |*β* − *β*_*R*_0__| < *ϵ* such that the signs of these coefficients are preserved. Although in this case we do not have the solution *x* = 0, we eventually could still have two positive solutions and consequently, multiple equilibrium states; see the green line in Figure 3. 

*Example II*. Suppose we take numerical values for the parameters in Table 1 such that the condition *β*_*B*_ < *β*_*C*_ < *β*_*R*_0__ is fulfilled.

If *β* < *β*_*B*_, then all coefficients of the polynomial (20) are positive and there is not nonnegative solutions. In this case, the system has only a disease-free equilibrium.

For *β*_*B*_ < *β* < *β*_*C*_ and *β*_*C*_ < *β* < *β*_*R*_0__ the signs of the coefficients of the polynomial are *A* > 0, *B* < 0, *C* > 0, and *D* > 0, *A* > 0, *B* < 0, *C* < 0, *D* > 0, respectively.

In both cases the polynomial has two possibilities:

- three real solutions: one negative and two positive solutions for Δ_1_ < 0,
- one negative and two complex conjugate solutions for Δ_1_ > 0.

Here Δ_1_ is the discriminant for the polynomial (20).

In the (a) case we have the possibility of multiple endemic states for system (1). This case is illustrated in numerical simulations in the next section by Figures 8 and 9.

We should note that the value *β* = *β*_*B*_ is not a bifurcation value for the parameter *β*.

If *β* = *β*_*B*_, then *A* > 0, *B* = 0, *C* > 0, and *D* > 0. In this case we have

(23) $$\begin{array}{c} \Delta_{1}=\frac{1}{4}\;\frac{D^{2}}{A^{2}}+\frac{1}{27}\;\frac{C^{3}}{A^{3}}>0. \end{array}$$

The discriminant Δ_1_ is a continuous function of *β*, for this reason this sign will be preserved in a *ϵ* neighbourhood of *β*_*B*_.

**Figure 4:**
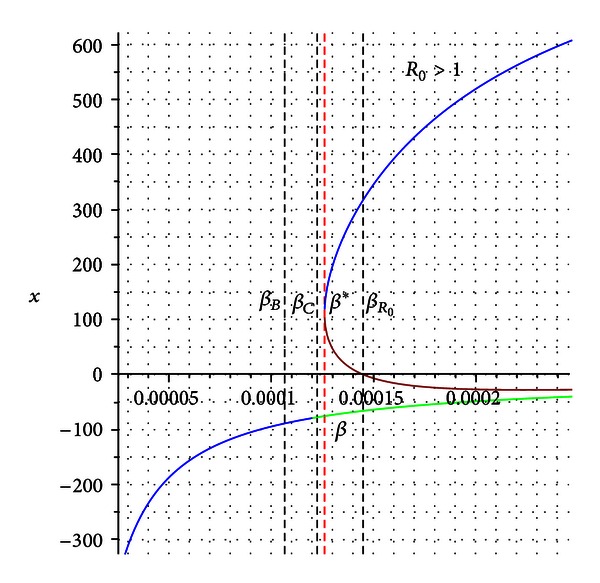
Bifurcation diagram for the condition *β*_*B*_ < *β*_*C*_ < *β*_*R*_0__. β* is the bifurcation value. The blue branch in the graph is a stable endemic equilibrium which appears even for *R*_0_ < 1.

We should be able to find a bifurcation value solving numerically the equation

(24) $$\begin{array}{c} \Delta_{1}\left(\beta^{*}\right)=0, \end{array}$$

where β* can be bounded by the interval *β*_*B*_ < β* < *β*_*R*_0__ (see Figure 4).

## 4. Numerical Simulations

In this section we will show some numerical simulations with the compartmental model (1). This model has fourteen parameters that have been gathered in Table 1. In order to make the numerical exploration of the model more manageable, we will adopt the following strategy.

- First, instead of fourteen parameters we will reduce the parametric space using four independent parameters *β*_*R*_0__, *β*, *δ*, and *η*. The parameters *β*, *δ*, and *η* are the transmission rate of primary infection, exogenous reinfection rate of latently infected, and exogenous reinfection rate of recovered individuals, respectively. *β*_*R*_0__ is the value of *β* such that basic reproduction number *R*_0_ is equal to one (or the value of *β* such that coefficient *D* in the polynomial (20) becomes zero). On the other hand, *β*_*R*_0__ depends on parameters given in the list Λ = {Π, *c*, *f*, *μ*, *ν*, *p*, *q*, *w*, *μ*_*t*_, *r*_1_, *r*_2_}. This means that if we keep all the parameters fixed in the list Λ, then *β*_*R*_0__ is also fixed. In simulations we will use *β*_*R*_0__ instead of using basic reproduction number *R*_0_.
- Second, we will fix parameters in the list Λ according to the values reported in the literature. In Table 4 are shown numerical values that will be used in some of the simulations, besides the corresponding references from where these values were taken. Mostly, these numerical values are related to data obtained from the population at large, and in the next simulations we will change some of them for considering the conditions of extremely high incidence/prevalence of TB in semiclosed communities. In any case, these changes will be clearly indicated in the text.
- Third, for any pairs of values *δ* and *η* we can compute *β*_*B*_ and *β*_*C*_, that is, the values of *β* such that *B* = 0 and *C* = 0, respectively, in the polynomial (20). So, we have that the exploration of parametric space is reduced at this point to the study of the parameters *β*_*R*_0__, *β*_*B*_, *β*_*C*_, and *β*. According to the chosen values for *δ*, *η*, and *β*_*R*_0__, we have six possible orderings for the parameters *β*_*R*_0__, *β*_*B*_, and *β*_*C*_ (see Appendix B).The dynamic behavior of system (1) will depend of these orderings. In particular, from Table 5, it is easy to see that if *β* ≤ min⁡(*β*_*R*_0__, *β*_*B*_, *β*_*C*_) then the system has a unique equilibrium point, which represents a disease-free state, and if *β* ≥ max⁡(*β*_*R*_0__, *β*_*B*_, *β*_*C*_), then the system has a unique endemic equilibrium, besides an unstable disease-free equilibrium.
- Fourth and finally, we will change the value of *β*, which is considered a bifurcation parameter for system (1), taking into account the previous mentioned ordering to find different qualitative dynamics.

It is especially interesting to explore the consequences of modifications in the values of the reinfection parameters without changing the values in the list Λ, because in this case the threshold *β*_*R*_0__ remains unchanged. Thus, we can study in a better way the influence of the reinfection in the dynamics of the TB spread.

The values given for the reinfection parameters *δ* and *η* in the next simulations could be extreme, trying to capture this way the special conditions of high burden semiclosed communities.

*Example I (Case β*_*R*_0__ < *β*_*C*_ < *β*_*B*_,  *δ* = 0.9, *η* = 0.01*).* Let us consider here the case when the condition *β*_*R*_0__ < *β*_*C*_ < *β*_*B*_ is met. We know from the previous section that this condition is met under biologically plausible values (*δ*, *η*)∈[0,1]×[0,1].

According to Lemmas 3 and 4, in this case the behaviour of the system is characterized by the evolution towards disease-free equilibrium if *β* < *β*_*R*_0__ and the existence of a unique endemic equilibrium for *β* > *β*_*R*_0__. Changes in the parameters of the list Λ alter the numerical value of the threshold *β*_*R*_0__ but do not change this behaviour.

First, we consider the following numerical values for these parameters: *δ* = 0.9, *η* = 0.01, and *β* = 0.00052. We also fix the list of parameters Λ according to the numerical values given in Table 4.

The basic reproduction number for these numerical values gives *R*_0_ = 3.585422172. The initial conditions considered were

(25) $$\begin{array}{c} S\left(0\right)=4980,\;\;E\left(0\right)=0,\;\;I_{I}\left(0\right)=20,\\ I_{N}\left(0\right)=0,\;\;R\left(0\right)=0. \end{array}$$

We also have the following values:

(26) $$\begin{array}{c} \beta_{R_{0}}=0.0001450317354,\\ \beta_{B}=0.01087387065,\\ \beta_{C}=0.0002715343808. \end{array}$$

These values clearly meet the condition *β*_*R*_0__ < *β*_*C*_ < *β*_*B*_, and according to Lemma 3 the system must have in this case a unique endemic equilibrium for all *β* > *β*_*R*_0__.

**Figure 5:**
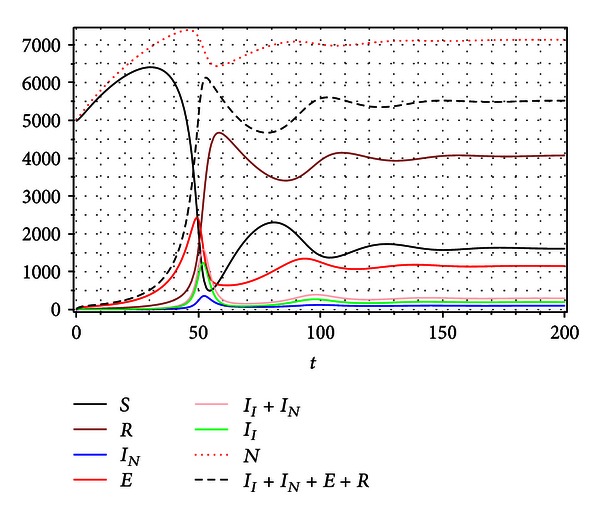
Numerical simulation for *R*_0_ = 3.585422172, *δ* = 0.9, *η* = 0.01, and *β* = 0.00052. The system goes toward a focus type stable stationary equilibrium.

Figure 5 shows that under the above described situation, the system will converge to an *endemic equilibrium* given by the *focus* type stationary steady solution:

(27) $$\begin{array}{c} S_{\infty }=1616,\;\;R_{\infty }=4080,\;\;I_{N\infty }=103,\\ I_{I\infty }=195,\;\;E_{\infty }=1150. \end{array}$$

By straightforward calculations we can show that this focus is stable, and no matter what initial conditions are taken for the system, the solutions always evolve to this endemic state.

**Figure 6:**
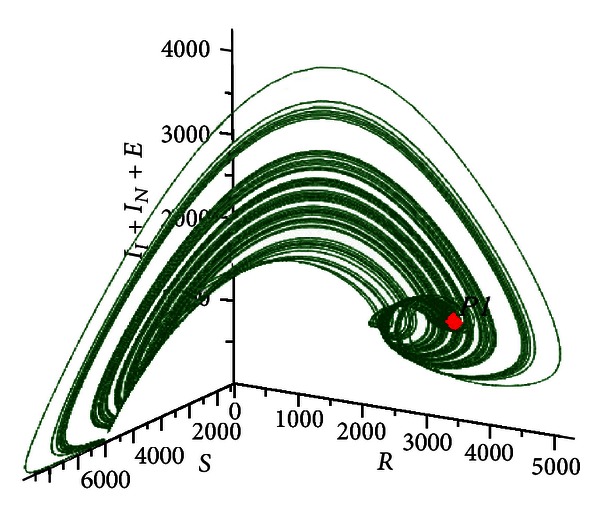
Phase space representation of the evolution of the system toward a stable focus type equilibrium. In this representation were used multiple initial conditions and the following values: *R*_0_ = 3.585422172, *δ* = 0.9, *η* = 0.01, and *β* = 0.00052.

Figure 6 shows the trajectories of the system for multiple initial conditions in a three-dimensional phase space in which the horizontal axes are susceptible *S* and recovered *R* individuals, while the vertical axis is the prevalence *I*_*I*_ + *I*_*N*_ + *E*.

*Example II (Case β*_*R*_0__ < *β*_*C*_ < *β*_*B*_,  *δ* = 0.0, *η* = 0.9*).*  For our next numerical simulation we consider the following values for the used parameters: *δ* = 0.01, *η* = 0.9, *β* = 0.00052, and as before the list of parameters Λ is fixed according to Table 4.

The basic reproduction number for these parameters as before gives the same value *R*_0_ = 3.585422172. The used initial conditions were

(28) $$\begin{array}{c} S\left(0\right)=4980,\;\;E\left(0\right)=0,\;\;I_{I}\left(0\right)=20,\\ I_{N}\left(0\right)=0,\;\;R\left(0\right)=0. \end{array}$$

We also have the following values:

(29) $$\begin{array}{c} \beta_{R_{0}}=0.0001450317354,\\ \beta_{B}=0.01226355348,\\ \beta_{C}=0.0003132229272. \end{array}$$

These values meet the condition *β*_*R*_0__ < *β*_*B*_ < *β*_*C*_, and as in the previous simulation the system evolves toward a unique endemic equilibrium, but this time the dynamical properties of the equilibrium have changed.

**Figure 7:**
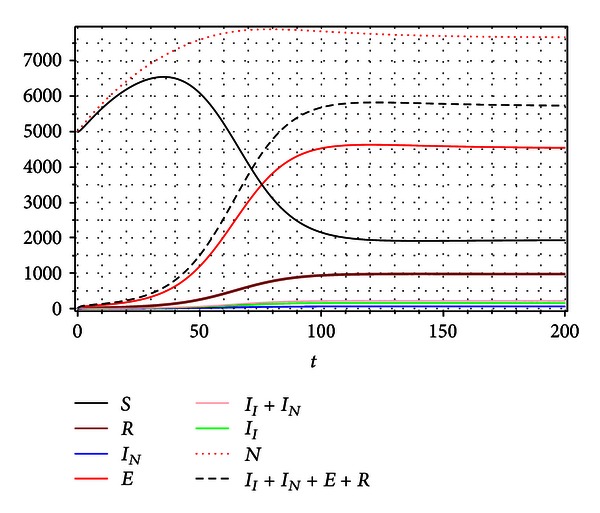
Numerical simulation for *R*_0_ = 3.585422172, *δ* = 0.01, *η* = 0.9, and *β* = 0.00052. In this case the system converges to a stable node type equilibrium.

In fact, Figure 7 shows the evolution of the system toward a stable *node* type endemic equilibrium:

(30) $$\begin{array}{c} S_{\infty }=1938,\;\;R_{\infty }=974,\;\;I_{N\infty }=60,\\ I_{I\infty }=156,\;\;E_{\infty }=4530. \end{array}$$

In our model, considering biologically plausible domain for exogenous reinfection parameters (*δ*, *η*)∈[0,1]×[0,1], the condition *β*_*R*_0__ < *β*_*C*_ < *β*_*B*_ is fulfilled. Under this condition we have a unique endemic equilibrium for *β* > *β*_*R*_0__. The emergence by a transcritical bifurcation of this endemic state is properly explained by the basic reproduction number *R*_0_. However, changes in the reinfection parameters *δ*, *η* can modify the qualitative nature of the dynamics of the disease, in addition to changing the numbers of individuals in the different compartments of the model in the endemic equilibrium state, without having any change in the value of the basic reproduction number *R*_0_, which in this case fails to describe these variations in the dynamics of the disease.

*Example III (Case β*_*B*_ < *β*_*C*_ < *β*_*R*_0__, *δ* = 3.0, *η* = 2.5*).*  There is now evidence that rates of secondary tuberculosis in high endemic communities (for example semiclosed communities), in patients with LTB or/and already treated for primary disease, are actually higher than in people presenting with primary infection [21, 22]. Taking this into consideration we consider now the following numerical values for the parameters: *β* = 0.00014, *δ* = 3.0, *η* = 2.5. In this case the basic reproduction number takes the value *R*_0_ = 0.9653059690. Additionally we have

(31) $$\begin{array}{c} \beta_{R_{0}}=0.0001450317354,\\ \beta_{B}=0.0001066568066,\\ \beta_{C}=0.0001225687204. \end{array}$$

For these parameter we have that the condition *β*_*B*_ < *β*_*C*_ < *β*_*R*_0__ is fulfilled and the system has the possibility of multiple equilibria. In fact, we have in this case the following stationary points *P* = (*S*, *R*, *I*_*i*_, *I*_*n*_, *E*):

(32) $$\begin{array}{c} P_{1}=\left(9009,0,0,0,0\right),\\ P_{2}=\left(8507,182,9,5,2166\right),\\ P_{3}=\left(3221,1406,285,103,1566\right). \end{array}$$

*P*_1_ is a stable disease-free equilibrium point (stable node), *P*_3_ is a stable endemic equilibrium (stable focus), and *P*_2_ is an unstable equilibrium point (saddle point).

**Figure 8:**
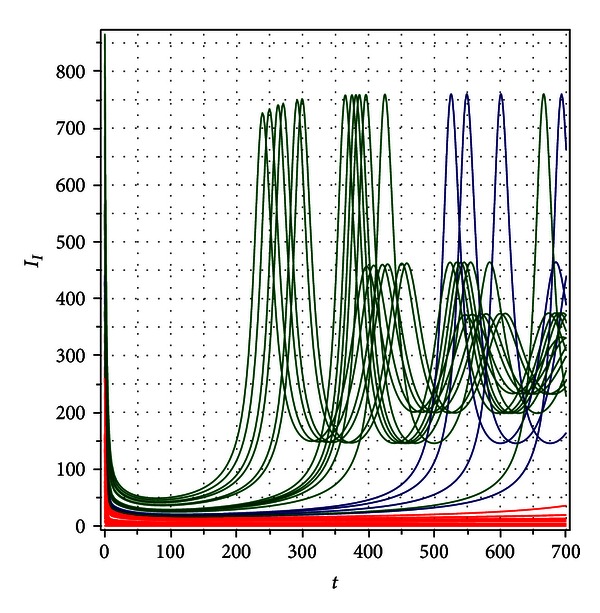
Numerical simulation for *R*_0_ = 0.9653059690, *δ* = 3.0, and *η* = 2.5. The system can evolve to two different equilibria *I*_*I∞*_ = 0 (red lines) or *I*_*I∞*_ = 285 (dark green lines) according to different initial conditions.

Figure 8 shows the convergence to *I*_*I∞*_ = 0 or to *I*_*I∞*_ = 285 according to with different initial conditions.

**Figure 9:**
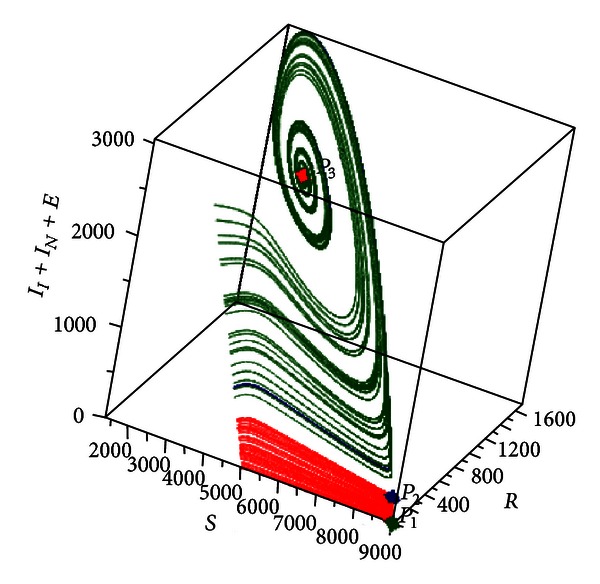
Numerical simulation for *R*_0_ = 0.9653059690, *δ* = 3.0, and *η* = 2.5. Phase space representation of the system with multiple equilibrium points.

In Figure 9 is shown another representation (phase space) of the evolution of the system toward *P*_1_ or to *P*_3_ according to different initial conditions. The representation is a three-dimensional phase space in which the horizontal axes are susceptible *S* and recovered *R* individuals, while the vertical axis is the prevalence *I*_*I*_ + *I*_*N*_ + *E*.

For the previously numerical values, the system experiences a *backward bifurcation* [37] at the value *β*_∗_ = 0.0001261648723 with *β*_*B*_ < *β*_∗_ < *β*_*R*_0__. For *β* > *β*_∗_, the system possesses two stable equilibrium points and one unstable (see Figure 4).

*Example IV (Case β*_*B*_ < *β*_*R*_0__ < *β*_*C*_, *δ* = 3.0, *η* = 2.5*).* Consider now a more extreme situation with *η* = 2.5, *δ* = 3.0, and *p* = 0.7 (the other parameters kept the same values given in Table 4). In this case the condition *β*_*B*_ < *β*_*R*_0__ < *β*_*C*_ is fulfilled.

This example is shown in order to illustrate more complex and rich dynamics that might admit system (1), which is mathematically possible and could in principle be a model case for an extreme hypothetical situation in a semiclosed high burden community. For these parameters we have

(33) $$\begin{array}{c} \beta_{R_{0}}=0.0001679568390,\\ \beta_{C}=0.0001729256777,\\ \beta_{B}=0.0001489092005, \end{array}$$

which clearly satisfy the condition *β*_*B*_ < *β*_*R*_0__ < *β*_*C*_. Therefore, as was explained in the previous section, the system has the possibility of multiple equilibria.

In fact, for the bifurcation value *β*_1_ = 0.0001673533706 of the disease transmission rate, which satisfies the condition *β*_*B*_ < *β*_1_ < *β*_*R*_0__, the system acquires two positive equilibria, apart from the disease-free equilibrium.

**Figure 10:**
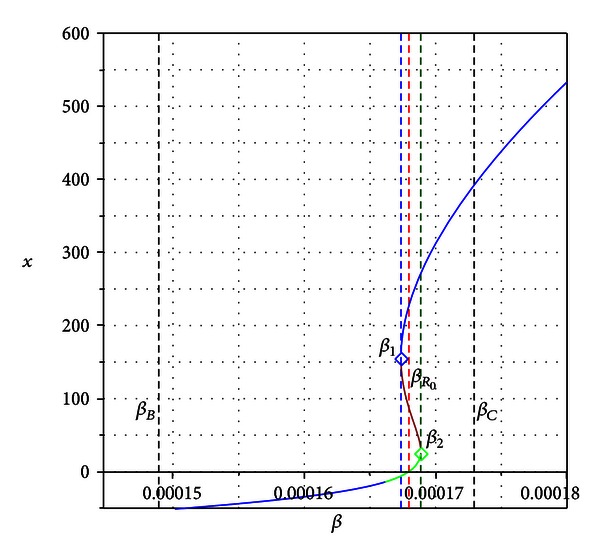
Bifurcation diagram (solution *x* of polynomial (20) versus *β*) for the condition *β*_*B*_ < *β*_*R*_0__ < *β*_*C*_. The system experiences multiple bifurcations at *β*_1_, *β*_*R*_0__, and *β*_2_.

When *β* = *β*_*R*_0__ appear three positive equilibrium points and the disease-free equillibrium becomes unstable. For *β*_2_ = 0.0001688612368 with *β*_*R*_0__ < *β*_2_ < *β*_*C*_ the system admits a unique and stable endemic equilibrium (see Figure 10).

We take now the value *β* = 0.0001675, which satisfies the condition *β*_1_ < *β* < *β*_*R*_0__.

With these numerical values the basic reproduction number is *R*_0_ = 0.9972800211 < 1, and therefore, the disease-free equilibrium is stable.

We have in this case the following stationary points *P* = (*S*, *R*, *I*_*i*_, *I*_*n*_, *E*):

(34) $$\begin{array}{c} P_{0}=\left(5148,0,0,0,0\right),\\ P_{1}=\left(3372,1041,122,60,482\right),\\ P_{2}=\left(2828,1283,190,88,651\right). \end{array}$$

**Figure 11:**
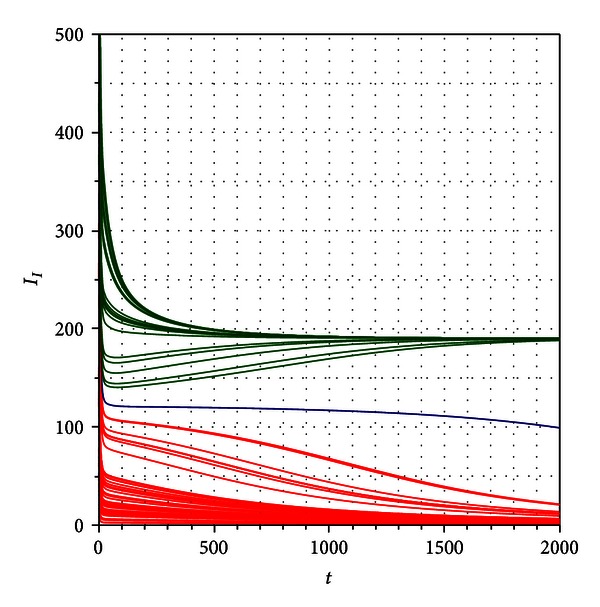
Numerical simulation for *R*_0_ = 0.9972800211, *δ* = 3.0, and *η* = 2.5. The system can evolve to two different equilibria *I*_*I∞*_ = 0 or *I*_*I∞*_ = 190 according to the initial condition.

*P*_0_ is the stable disease-free equillibrium point (stable node), *P*_1_ is an unstable equilibrium point (saddle point), and *P*_2_ is a stable endemic equilibrium (stable focus).  Figure 11 shows the convergence to *I*_*I∞*_ = 0 or to *I*_*I∞*_ = 190 according to the initial condition.

**Figure 12:**
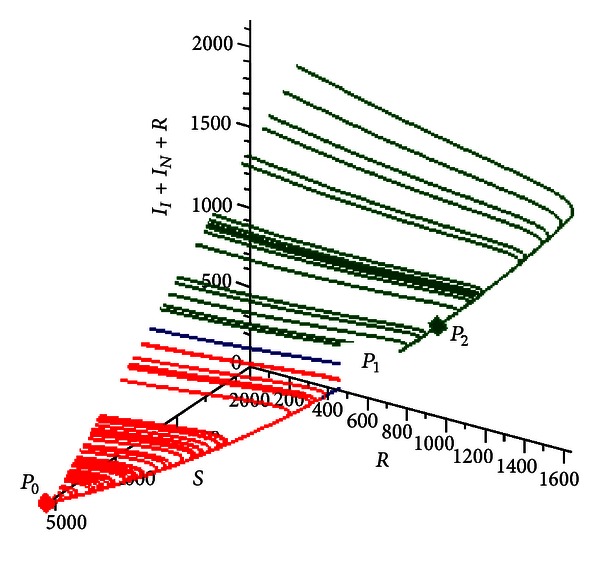
Numerical simulation for *R*_0_ = 0.9972800211, *δ* = 3.0, and *η* = 2.5. Phase space representation of the system with multiple equilibrium points.

In Figure 12 is shown another representation (phase space) of the evolution of the system toward *P*_0_ or to *P*_2_ according to the initial conditions.

Let us take now the value *β* = 0.0001683, which satisfies the condition *β*_*R*_0__ < *β* < *β*_2_. In this case, the basic reproduction number has the value *R*_0_ = 1.002043150. We still have that the condition *β*_*B*_ < *β*_*R*_0__ < *β*_*C*_ is fulfilled and the system in this case has four equilibrium points *P* = (*S*, *R*, *I*_*i*_, *I*_*n*_, *E*):

(35) $$\begin{array}{c} P_{0}=\left(5148,0,0,0,0\right),\\ P_{1}=\left(5042,76,5,3,20\right),\\ P_{2}=\left(3971,734,69,36,298\right),\\ P_{3}=\left(2491,1413,246,109,750\right). \end{array}$$

*P*_0_ is the unstable disease-free equillibrium point (saddle point ), *P*_1_ is a stable endemic equilibrium point (node), *P*_2_ is an unstable equilibrium (saddle point), and *P*_3_ is a stable endemic equilibrium point (focus).

**Figure 13:**
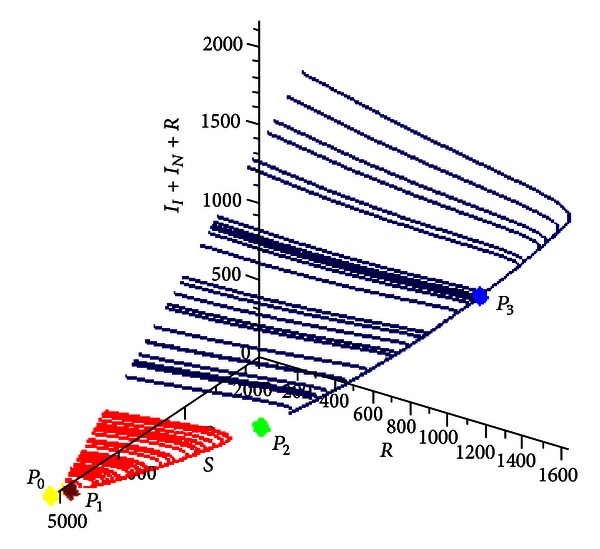
Numerical simulation for *R*_0_ = 1.002043150, *δ* = 3.0, and *η* = 2.5. The system can evolve to two different equilibria *P*_1_ (stable node) or *P*_3_ (stable focus) according to the initial condition. *P*_0_ and *P*_2_ are unstable equilibria.

Figure 13 shows the phase space representation of this case.

For further numerical analysis, we set all the parameters in the list Λ according to the numerical values given in Table 4, leaving *free* the parameters *β*, *η*, and *δ* related to the primary transmission rate and reinfection rates of the disease.

We will explore the parametric space of system (1) and relate it to the signs of the coefficients of the polynomial (20).

**Figure 14:**
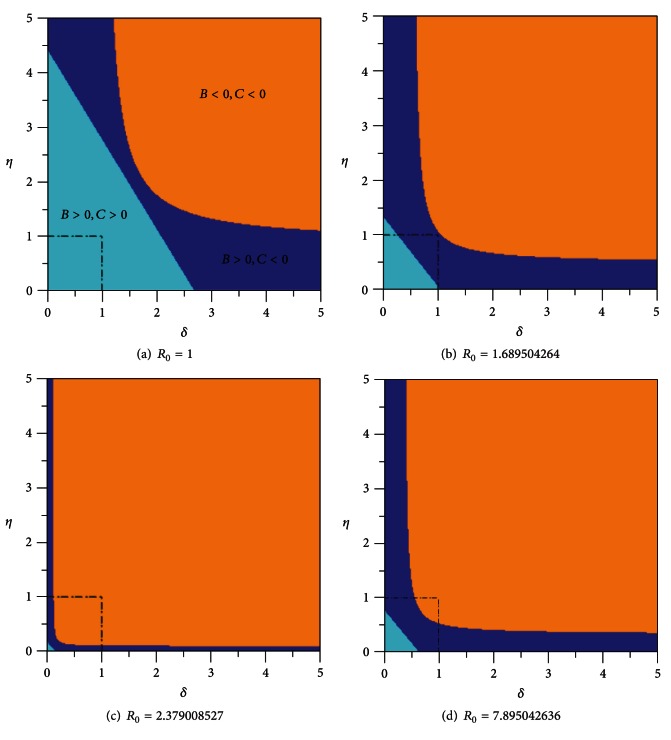
Signs of coefficients *B* and *C* as functions of exogenous reinfection rate of latent *δ* and exogenous reinfection rate of recovered *η* for *R*_0_⩾1. The parameter *β* has the values: (a) *β* = *β*_*R*_0__ = 0.0001450317354, (b) *β* = 0.0002450317354, (c) *β* = 0.0003450317354, and (d) *β* = 0.001145031735.

In Figure 14, we consider values of *β* such that *R*_0_ > 1. We can observe from this figure that as the primary transmission rate of the disease *β* increases, and with it the basic reproduction number *R*_0_, the system under biological plausible condition, represented in the figure by the square (*δ*, *η*) ∈ [0,1] × [0,1], evolves such that initially (for lower values of *β*) coefficients *B* and *C* are both positive, then *B* remains positive and *C* becomes negative and finally both coefficients become negative.

This change in the coefficients signs as the transmission rate *β* increases agrees with the results summarized in Table 2 when the condition *β*_*R*_0__ < *β*_*C*_ < *β*_*B*_ is fulfilled.

Next, in order to explore another mathematical possibilities we will modify some numerical values for the parameters in the list Λ in a more extreme manner, taking a hypothetical regime with Λ* = {*μ* = 0.03885, *μ*_*t*_ = 0.01520, *p* = 0.8, *ν* = 0.0266, *f* = 0.8, *q* = 0.85, *w* = 0.005, *c* = 0.4, *r*_1_ = 0.5, *r*_2_ = 0.2}.

**Figure 15:**
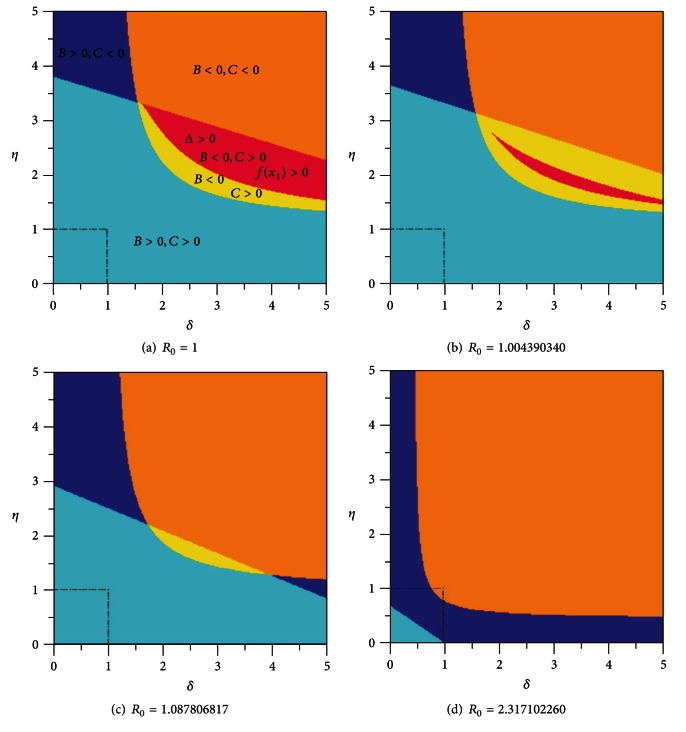
Signs of coefficients *B*, *C*, and discriminant Δ = *B* ^2^ − 3*AC* as functions of exogenous reinfection rate of latent *δ* and exogenous reinfection rate of recovered *η* for *R*_0_⩾1. The parameter *β* has the values: (a) *β* = *β*_*R*_0__ = 0.0002277727471, (b) *β* = 0.0002287727471, (c) *β* = 0.0002477727471, and (d) *β* = 0.0005277727471.

In Figure 15 besides signs of *B* and *C* we consider also the signs of the discriminant Δ of the quadratic equation *P*′(*x*) = 0, where *P*(*x*) is the polynomial (20).

From Figure 15, in particular we can see that the domain with *B* < 0, *C* > 0, Δ > 0, and *P*(*x*_1_) > 0 represented in red allows the possibility of multiple endemic equilibria for system (1). However, despite the extreme numerical values for the parameters taking in Λ*, this domain is still far from the domain of biologically plausible values for *δ* and *η*, represented in the figures by the square (*δ*, *η*)∈[0,1]×[0,1].

As the transmission rate of the disease *β* increases, and with it the basic reproduction number *R*_0_, this red domain of the parametric space becomes increasingly smaller until finally it disappears. In fact, the red domain is only significant when the basic reproduction number *R*_0_ is near one.

**Figure 16:**
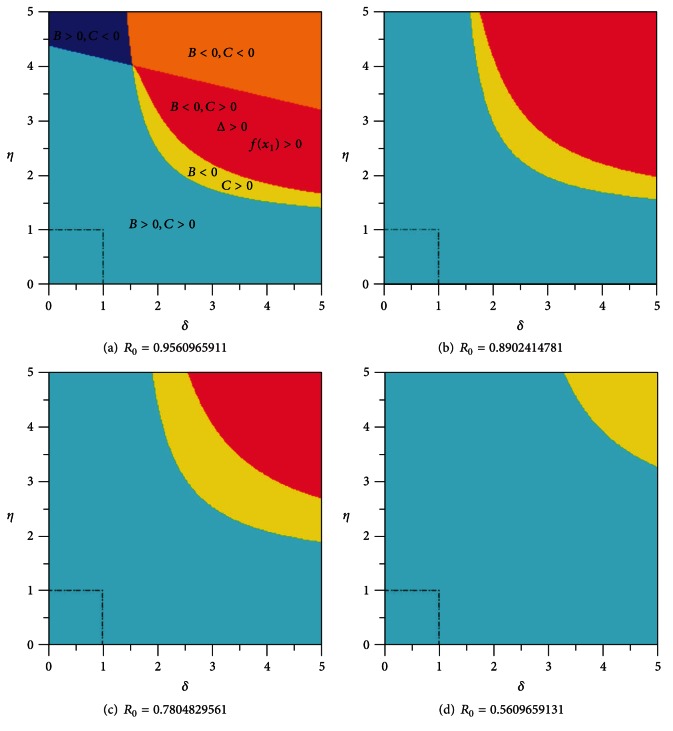
Signs of coefficients *B*, *C*, and discriminant Δ = *B* ^2^ − 3*AC* as functions of exogenous reinfection rate of latent *δ* and exogenous reinfection rate of recovered *η* for *R*_0_⪕1. The parameter *β* has the values: (a) *β* = 0.0002177727471, (b) *β* = 0.0002027727471, (c) *β* = 0.0001777727471, and (d) *β* = 0.0001277727471.

In Figure 16, we show some numerical simulation where basic reproduction number *R*_0_ is less than one. The red domain indicates the possibility of multiple endemic equilibria for the system even for *R*_0_ < 1. We can see that this domain is far from the domain of biologically plausible values for *δ* and *η* represented in the figure by the square (*δ*, *η*)∈[0,1]×[0,1]. As the transmission rate of the disease *β* decreases, and with it the number *R*_0_, this parameter domain moves away from the square. In all represented cases *B* > 0 and *C* > 0 inside the square (*δ*, *η*)∈[0,1]×[0,1].

## 5. Discussion and Conclusions

In order to consider high incidence and prevalence of TB and LTB in semiclosed communities, we have used in this work a compartmental SEIR model with five possible pathways to TB disease. The extra nonlinear terms considered in the model lead to a more complex mathematical treatment in comparison with previously used models (see e.g., [23, 25–29, 32, 35, 36]). But the special form of some coefficients obtained from the analysis of standard SEIR models with constant transmission rate allowed us to move forward with some analytical results that were confirmed later by numerical simulations.

In this paper we follow a new methodology for numerical exploration of this kind of models, which allowed us to handle the high dimensionality of its parameter spaces and thus study its different dynamic behaviors. We found that the transmission rate *β* can be used as a bifurcation parameter and that the system undergoes qualitative changes in the dynamics when this parameter varies. In this context the analysis of the parametric space is reduced to the study of variations of the parameters *β*_*R*_0__, *β*_*B*_, *β*_*C*_, and *β*. We divided the parametric space into six possible arrangements for these parameters, which in turn determine all possible different qualitative behaviours of the system dynamics.

From model (1) we can see that reinfection requires latently infected, recovered, and actively infectious individuals. The basic reproduction number *R*_0_ has to do solely with infections produced when an infectious individual is introduced into an uninfected population, but reinfection does not alter the dynamics of an uninfected population. So, the number *R*_0_ does not completely describe the dynamics of the model when the reinfection is incorporated, as was noted before by Feng et al. in [26]. Unlike the model published by these authors, which uses a single parameter for exogenous reinfection, in our model we use two parameters related to two possible pathways of reinfection (reinfection of latently infected and reinfection of recovered individuals).This is a reason why our model shows a more complex and richer dynamics.

We have showed through theoretical analysis in Section 3 and numerical simulations in Section 4 that if we accept as valid the plausible assumption that exposure to mycobacterium induces an immune response, which is partially protective against reinfection, then the system for semiclosed communities (1) reproduces well, common observed trends in TB epidemiology that are similar to what happens in population at large, which is basically that, for *R*_0_ < 1, there is only one disease-free status, while for *R*_0_ > 1, there exists a unique endemic state with nonzero prevalence. For *R*_0_ = 1 occurs a transcritical bifurcation from which emerges an endemic stable state.

Moreover, according to Lemmas 3 and 4, any values of reinfection parameters in this parametric regime: (*δ*, *η*)∈[0,1]×[0,1] would lead to the same qualitative dynamics and will not affect this already classical behavior in SEIR models. In this case only one of the aforementioned arrangements (*β*_*R*_0__ < *β*_*C*_ < *β*_*B*_) emerges as valid under this biologically plausible condition.

Since the two parameters related to exogenous reinfection of latently infected and recovered individuals do not affect the value of the number *R*_0_, even under the plausible assumption of partial immunity, variation of reinfection parameters can make that for the same value of the number *R*_0_, the quality of dynamics and the number of affected by disease individuals (incidence and prevalence) drastically change. For example, Figures 5 and 7 show two types of dynamics, that is, convergences to different stationary points, a focus and a node for the same basic reproduction number *R*_0_. Some evidence of this variability in tuberculosis epidemiology due to dynamic balance between primary infection and reinfection has been presented in several works (see e.g., [26, 38]).

Taking less plausible assumption, but already evidenced in several works [5, 21, 22, 26], of an increased susceptibility to reinfection over primary infection in some cases leads us to a further study of model (1). For *δ* > 1 and *η* > 1 system (1) experiences a rich and complex dynamics with successive and different kind of bifurcations as the transmission rate *β* changes. These cases incorporate possible multiple endemic states, regardless of whether the values for the basic reproduction number *R*_0_ were less than or greater than 1. So, these behaviors cannot be explained using only this number. It is in this context that the use of the disease transmission rate *β* as bifurcation parameter instead of *R*_0_ acquires real usefulness.

Some important implications of the simulations with *δ* > 1 and *η* > 1 lie in the fact that many of the measures taken to stop and control an epidemics are designed to reduce the value of the basic reproduction number *R*_0_ such that disease-free status for *R*_0_ < 1 is achieved. However, in this parametric regime, reinfection might cause the system to fall into a state unable to eliminate endemic disease, although it fulfills that *R*_0_ < 1. Thus, semiclosed communities with this kind of regime will become in genuine high transmission pockets of TB inserted in the general population [4]. Indeed, semiclosed communities such as prisons might become in a reservoir for disease transmission to the population at large and should be a source of public concern [4, 6, 7].

The theoretical approach and numerical simulations presented in this paper for the study of the impact of reinfection on TB dynamics in semiclosed communities could have important implications at multiple levels, including vaccine design, control program design, epidemiology of tuberculosis in regions where the risk of reexposure is high, and for systems-based computer models which to date assume that primary infection will confer at least some degree of (stable) memory immunity to a secondary infection, but that in fact also have to consider less plausible assumptions about an increased susceptibility to reinfection.

## Figures and Tables

**Table 1:** Parameters of the model, its descriptions, and its units.

Parameter	Description	Unit
*β*	Transmission rate	1/year
Π	Recruitment rate	1/year
*c*	Natural cure rate	1/year
*ν*	Progression rate from latent TB to active TB	1/year
*μ*	Natural mortality rate	1/year
*μ*_*T*_	Mortality rate or fatality rate due to TB	1/year
*w*	Relapse rate	1/year
*q*	Probability to develop TB (slow case)	—
*f*	Probability to develop TB (fast case)	—
*p*	Proportion of new infections that produce active TB	—
*δβ*	Exogenous reinfection rate of latent	1/year
*ηβ*	Exogenous reinfection rate of recovered	1/year
*r*_1_	Treatment rates for *I*_*I*_	1/year
*r*_2_	Treatment rates for *I*_*N*_	1/year

**Table 2:** Qualitative behaviour for system (1) as a function of the disease transmission rate *β*, when the condition β_R_0__ < β_C_ < *β*_*B*_ is fulfilled.

Interval	Coefficients	Type of equilibrium
*β* < *β*_*R*_0__	*A* > 0, *B* > 0, *C* > 0, *D* > 0	Disease-free equilibrium
*β*_*R*_0__ < *β* < *β*_*C*_	*A* > 0, *B* > 0, *C* > 0, *D* < 0	Unique endemic equilibrium
*β*_*C*_ < *β* < *β*_*B*_	*A* > 0, *B* > 0, *C* < 0, *D* < 0	Unique endemic equilibrium
*β* > *β*_*B*_	*A* > 0, *B* < 0, *C* < 0, *D* < 0	Unique endemic equilibrium

**Table 3:** Qualitative behaviour for system (1) as function of the disease transmission rate *β*, when the condition *β*_*B*_ < *β*_*C*_ < *β*_*R*_0__ is fulfilled. Here, Δ_1_ is the discriminant of the cubic polynomial (20).

Interval	Coefficients	Type of equilibrium
*β* < *β*_*B*_	*A* > 0, *B* > 0, *C* > 0, *D* > 0	Disease-free equilibrium
*β*_*B*_ < *β* < *β*_*C*_	*A* > 0, *B* < 0, *C* > 0, *D* > 0	Two equilibria (Δ_1_ < 0) or none (Δ_1_ > 0)
*β*_*C*_ < *β* < *β*_*R*_0__	*A* > 0, *B* < 0, *C* < 0, *D* > 0	Two equilibria (Δ_1_ < 0) or none (Δ_1_ > 0)
*β*_*R*_0__ < *β*	*A* > 0, *B* < 0, *C* < 0, *D* < 0	Unique endemic equilibrium

**Table 4:** Numerical values for the parameters in the list Λ. Some of the given numerical values for the model parameters are mainly related to the spread of TB in the population at large and are basically taken as reference. Other values assuming for the parameters, different than those given in this table will be clearly indicated in the text.

Parameter	Description	Value
Π	Recruitment rate	200 (assumed)
*c*	Natural cure rate	0.058 [23, 33, 34]
*ν*	Progression rate from latent TB to active TB	0.0256 [33, 34]
*μ*	Natural mortality rate	0.0222 [2]
*μ*_*T*_	Mortality rate due to TB	0.139 [2, 33]
*w*	Relapse rate	0.005 [2, 33, 34]
*q*	Probability to develop TB (slow case)	0.85 [2, 33]
*f*	Probability to develop TB (fast case)	0.70 [2, 33]
*p*	Proportion of new infections that produce active TB	0.05 [2, 33, 34]
*r*_1_	Treatment rates for *I*_*I*_	0.50 (assumed)
*r*_2_	Treatment rates for *I*_*N*_	0.20 (assumed)

**Table 5:** Different possible orderings for *β*_*R*_0__, *β*_*B*_, and *β*_*C*_. In every case *A* > 0, Δ_1_ is the cubic discriminant of the equation *P*(*x*) = 0, Δ is the discriminant of the quadratic equation *P*′(*x*) = 0, where *P*(*x*) is the polynomial (20).

Interval	Coefficients	Equilibria
*β*_*R*_0__ < *β*_*C*_ < *β*_*B*_		

*β* < *β*_*R*_0__	*B* > 0, *C* > 0, *D* > 0	Disease-free equilibrium
*β*_*R*_0__ < *β* < *β*_*C*_	*B* > 0, *C* > 0, *D* < 0	Unique endemic equilibrium
*β*_*C*_ < *β* < *β*_*B*_	*B* > 0, *C* < 0, *D* < 0	Unique endemic equilibrium
*β*_*B*_ < *β*	*B* < 0, *C* < 0, *D* < 0	Unique endemic equilibrium

*β*_*C*_ < *β*_*R*_0__ < *β*_*B*_		

*β* < *β*_*C*_	*B* > 0, *C* > 0, *D* > 0	Disease-free equilibrium
*β*_*C*_ < *β* < *β*_*R*_0__	*B* > 0, *C* < 0, *D* > 0	Two equilibria if *P*(*x*_2_) ≤ 0 or Δ_1_ < 0; none if *P*(*x*_2_) ≥ 0 or Δ_1_ > 0
*β*_*R*_0__ < *β* < *β*_*B*_	*B* > 0, *C* < 0, *D* < 0	One equilibrium for Δ_1_ < 0 or Δ_1_ > 0
*β*_*B*_ < *β*	*B* < 0, *C* < 0, *D* < 0	Unique endemic equilibrium

*β*_*C*_ < *β*_*B*_ < *β*_*R*_0__		

*β* < *β*_*C*_	*B* > 0, *C* > 0, *D* > 0	Disease-free equilibrium
*β*_*C*_ < *β* < *β*_*B*_	*B* > 0, *C* < 0, *D* > 0	Two equilibria if *P*(*x*_2_) ≤ 0 or Δ_1_ < 0; none if *P*(*x*_2_) ≥ 0 or Δ_1_ > 0
*β*_*B*_ < *β* < *β*_*R*_0__	*B* < 0, *C* < 0, *D* > 0	Two equilibria (Δ_1_ < 0) or none (Δ_1_ > 0)
*β*_*R*_0__ < *β*	*B* < 0, *C* < 0, *D* < 0	Unique endemic equilibrium

*β*_*R*_0__ < *β*_*B*_ < *β*_*C*_		

*β* < *β*_*R*_0__	*B* > 0, *C* > 0, *D* > 0	Disease-free equilibrium
*β*_*R*_0__ < *β* < *β*_*B*_	*B* > 0, *C* > 0, *D* < 0	Unique endemic equilibrium
*β*_*B*_ < *β* < *β*_*C*_	*B* < 0, *C* > 0, *D* < 0	One equilibrium (Δ_1_ > 0), three equilibria (Δ_1_ < 0)
*β*_*C*_ < *β*	*B* < 0, *C* < 0, *D* < 0	Unique endemic equilibrium

*β*_*B*_ < *β*_*R*_0__ < *β*_*C*_		

*β* < *β*_*B*_	*B* > 0, *C* > 0, *D* > 0	Disease-free equilibrium
*β*_*B*_ < *β* < *β*_*R*_0__	*B* < 0, *C* > 0, *D* > 0	Two equilibria (Δ_1_ < 0) or none (Δ_1_ > 0)
*β*_*R*_0__ < *β* < *β*_*C*_	*B* < 0, *C* > 0, *D* < 0	One equilibrium (Δ_1_ > 0), three equilibria (Δ_1_ < 0)
*β*_*C*_ < *β*	*B* < 0, *C* < 0, *D* < 0	Unique endemic equilibrium

*β*_*B*_ < *β*_*C*_ < *β*_*R*_0__		

*β* < *β*_*B*_	*B* > 0, *C* > 0, *D* > 0	Disease-free equilibrium
*β*_*B*_ < *β* < *β*_*C*_	*B* < 0, *C* > 0, *D* > 0	Two equilibria (Δ_1_ < 0) or none (Δ_1_ > 0)
*β*_*C*_ < *β* < *β*_*R*_0__	*B* < 0, *C* < 0, *D* > 0	Two equilibria (Δ_1_ < 0) or none (Δ_1_ > 0)
*β*_*R*_0__ < *β*	*B* < 0, *C* < 0, *D* < 0	Unique endemic equilibrium

## Appendices

### A. Explicit Form of Coefficients

Introducing the notations:

(A.1) a=μ+μT+c,b=2w+μ,h=ν+μ,g=r1+c,m=r2+c,θ=μ+μt,ϵ=1−p,L1=θ+g(1−q)+mq,L2=νqr2+ha+r1ν(1−q)+r1μ,K1=r2w+ba+r1(w+μ),K2=νqr2+ha+r1ν(1−q)+r1μ.

We have

(A.2) B1=(θ(fp+(1−p)q)+mq)Πδσ,B2=σ(μL1θ  δ+L2θ+mμ(a+r1))+(r2wθ+r1(w+μ)θ+baθ+mμ(a+r1))δ,C1=Π(δ((fp+ϵq)(bμt+(m+b)μ)+mw)+σ((νqϵ+fhp)θ+m(μfp+ν  q))),C2=μ(K1θ+mμ(a+r1))δ+μ(K2θ+mμ(a+r1))σ+h(K1θ+mμ(a+r1)).

The coefficients *B* and *C* can be written in the following general form:

(A.3) $$\begin{array}{c} B=\beta^{2}f_{B}\left(\beta \right),\\ C=\beta f_{C}\left(\beta \right), \end{array}$$

where

(A.4) fB(β)=−B1β+B2,fC(β)=−C1β+C2,A=β3δσ(μ+μT)(μ+μT+c+(1−q)r1+qr2)>0,D=h([ab+wr2+r1(w+μ)](μ+μt)+μm(r1+a))(1−R0).

### B. Summary for Possible Orderings for the Different Transmission Rate Parameters

Table 5 shows different orderings for the parameters *β*_*R*_0__, *β*_*B*_, and *β*_*C*_ and their implications concerning the possible equilibrium or stationary points of system (1). This table is complemented with the analysis given in Section 3.1.2.
